# Real-life multicenter experience of long-term treatment with venetoclax plus azacitidine for acute myeloid leukemia in China

**DOI:** 10.1038/s41598-026-50426-0

**Published:** 2026-04-24

**Authors:** Xuexing Chen, Qiong Yan, Chunfang Li, Fei Liu, Shaojun Guo, Juan Zhao, Yan Liang, Haiyan Wang, Weiming Li, Guolin Yuan

**Affiliations:** 1 Department of Hematology, Xiangyang Central Hospital, Affiliated Hospital of Hubei University of Arts and Science, Xiangyang, 441000 China; 2 Department of Hematology, Wuhan Central Hospital, Affiliated Hospital of Huazhong, University of Science and Technology, Wuhan, China; 3 Department of Hematology, Jinzhou Central Hospital, Jinzhou, China; 4 Department of Hematology, Yichang Central Hospital, Yichang, China; 5 Department of Hematology, Union Hospital, Tongji Medical College, Huazhong University of Science and Technology, Wuhan, China

**Keywords:** Venetoclax, Acute myeloid leukemia, Long-term treatment, Efficacy, Prognostic factors, Cancer, Diseases, Medical research, Oncology

## Abstract

The combination of venetoclax (VEN) and azacitidine (AZA) has transformed the treatment of acute myeloid leukemia (AML) in patients ineligible for intensive therapy. Nevertheless, real-world evidence on long-term outcomes in patients with AML treated with VEN + AZA remains limited. A retrospective analysis was conducted on 79 patients with naïve AML who received at least 6 cycles of VEN + AZA across five collaborating hematology units in China between September 2021 and October 2024. The median age at diagnosis was 67 years (range: 49–87), and the median number of VEN treatment cycles was 9 (range: 6–20). The CR/CRi occurred in 56 of 79 patients (70.9%), with higher rates observed in those harboring NPM1 (80.9%) and IDH2 (80.0%) mutations. The median overall survival (OS) reached 33.5 months, with a median follow-up of 14 months and a median duration of response of 25.4 months. Patients aged over 65 demonstrated inferior OS compared with younger patients (median OS: 26.1 months and not reached, respectively). Patients with NPM1 and IDH2 mutations were associated with improved OS, although the difference did not reach statistical significance. Patients treated with a lower dose of VEN achieved superior OS compared to those receiving the standard dose (*P* = 0.032). Prolonged VEN + AZA therapy is effective for AML, achieving sustained remission in a subset of patients and supporting its role as a viable maintenance therapy in this population.

**Supplementary Information:**

The online version contains supplementary material available at 10.1038/s41598-026-50426-0.

## Introduction

Acute myeloid leukemia (AML) is a highly aggressive and heterogeneous myeloid neoplasm with a median age at diagnosis of 68 years^1^. Prognosis for older adults with AML or patients ineligible for intensive induction chemotherapy remains poor due to limited therapeutic options. Recently, the combination of venetoclax and azacitidine (VEN + AZA) has emerged as standard frontline treatment for this population^2^. Data from the phase 3 VIALE-A trial indicated that, after a median follow-up of 43 months, VEN + AZA arm achieved longer median overall survival (OS) (14.7 versus 9.6 months) and higher CR/CRi rates (67% versus 29%) compared with placebo plus AZA. Extended follow-up further demonstrated increased median duration of response (DOR) for CR/CRi (18.2 versus 17.5 months) in VEN + AZA arm, indicating sustained therapeutic benefit. However, hematologic adverse events of any grade occurred in 83% of patients receiving the VEN + AZA arm in VIALE-A trial, and most patients achieving CR/CRi required VEN dose and schedule adjustments to manage cytopenias during the long-term follow-up^3^. Compared with clinical trial data, evidence regarding long-term real-world use of VEN + AZA, particularly in the highly heterogeneous AML population, remains limited.

A retrospective multicenter analysis was conducted across five centers in China to evaluate the efficacy, survival outcomes, and treatment patterns (including daily VEN dosing and administration schedule) of VEN + AZA combination as long-term maintenance therapy for AML.

## Materials and methods

This multicenter observational registry included five public centers in China and was conducted from September 2021 to October 2024. Each center contributed data from electronic medical records to a centralized database with quality management. This study was approved by the XiangYang central hospital ethics committee and conducted in accordance with the Declaration of Helsinki. Informed consent was waived by XiangYang central hospital ethics committee. Review Board because of the retrospective nature of our study. A total of 120 patients with de novo AML were initially screened. Among them, 91 patients received induction therapy with azacitidine plus venetoclax (AZA + VEN), and 66 (72.5%) completed consolidation therapy with AZA + VEN at least 6 cycles. The remaining 29 patients received induction with AZA + VEN combined with chemotherapy(cytarabine, homoharringtonine, and G-CSF(CHG) or idarubicin, and cytarabine), of whom 13 (44.8%) completed at least 6 cycles. Ultimately, 79 patients were included in the final analysis. No concomitant antifungal agents were administered to any patient during VEN + AZA therapy. Baseline characteristics, including demographics, clinical and disease characteristics, treatment patterns, and outcomes, were analyzed descriptively. Response and risk stratification were assessed according to European LeukemiaNet (ELN) 2022 criteria^4^. Targeted sequencing was performed using a custom panel covering 45 genes recurrently mutated in myeloid malignancies in Supplementary Table S2. Overall survival (OS) for patients receiving AZA + VEN was calculated from therapy initiation to death or last follow-up. Relapse-free survival (RFS) was calculated from therapy initiation to relapse, last follow-up, or death, and OS was estimated from the initiation of therapy to last follow-up or death. OS was determined using the Kaplan–Meier method, and differences between groups were assessed using the log-rank test. To address potential immortal time bias in the comparison of patients with and without VEN dose reduction, a landmark analysis was performed. The landmark time point was set at the completion of cycle 2, as this represented the time by which the majority of dose reductions had occurred. For group comparisons, nominal variables were analyzed using the chi-square test or Fisher’s exact test, and continuous variables were evaluated using the Student’s t-test or Mann–Whitney U test, depending on data normality assessed by the Kolmogorov–Smirnov test. A Cox proportional hazards (PH) model analysis was performed to identify predictors of reduced OS and RFS. *P-*values < 0.05 were considered statistically significant. Analyses were conducted using the GraphPad Prism software (version 8).

## Results

### Baseline features

A total of 79 newly diagnosed AML patients treated with AZA + VEN from 2021 to 2024 were collected from five academic centers in China. Baseline and demographic characteristics of patients are summarized in Tables 1. The median age at diagnosis was 67 years (range: 49–87), with a predominance of male patients. According to ELN 2022 criteria, 19 patients (24.1%) had favorable risk, 34 (43.0%) had intermediate risk, and 26 (32.9%) had adverse risk. Interestingly, FAB-M5 was the most common subtype of leukemia, accounting for 49.4% of cases. The most common baseline mutations included IDH2 (27.8%), NPM1 (26.6%), DNMT3A (26.6%), and FLT3-ITD/TKD (21.3%).

**Table 1:** Baseline patient characteristics.

Variable	
Total number of patients	79
Median age (range), y	67 (49–87)
Sex	
Male/female	43/36
2022 ELN risk stratification, n (%)	
Favorable	19 (24.1%)
Intermediate	34 (43.0%)
Adverse	26 (32.9%)
Baseline WBC (× 109/L) Median (range)	5.53 (0.48–233)
Baseline platelet, (×109/L) Median (range)	48 (6−725)
Baseline bone marrow blast cell proportion	
< 30%	20
30%-50%	19
> 50%	49
FAB subtype	
M5	39 (49.4%)
M2	22 (27.8%)
M4	6 (7.6%)
M1/M0	4 (5.1%)
Unknown	8 (10.1%)
ECOG score	
0	20 (25.3%)
1	38 (48.1%)
>=2	21 (26.6%)
Selected molecular mutations	
NPM1	21 (26.6%)
FLT3-ITD/TKD	17 (21.3%)
DNMT3A	21 (26.6%)
CEBPA	8 (10.1%)
IDH2	22 (27.8%)
TET2	8 (10.1%)
SRSF2	5 (6.3%)
RUNX1	7 (8.9%)
BCOR	4 (5.1%)

### Treatment

Regarding induction chemotherapy, 66 patients received VEN + AZA regimen, 9 received VEN + AZA combined with CHG, and 4 received DVA regimen (venetoclax + idarubicin + cytarabine), followed by consolidation therapy with VEN + AZA from the second cycle. The median number of VEN treatment cycles was 9 (range: 6–20); 50 patients (63.3%) received a VEN target dose of 400 mg per day, and 29 (36.7%) received 200 mg per day. Most patients (63.3%) received VEN for 14 days, whereas 36.7% received the recommended 28-day duration, mainly determined by bone marrow evaluation and hematological toxicities (Table 2). Notably, patients treated with a lower VEN dose (< 400 mg) underwent significantly more cycles than those receiving the standard dose (median: 10 versus 7 cycles, respectively).

**Table 2:** Treatment details.

	*N* = 79
Median VEN cycles (range)	8 (6–20)
VEN dose (mg)	
200	29 (36.7%)
400	50 (63.3%)
VEN course (days)	
14/21	50 (63.3%)
28	29 (36.7%)

### Response and survival

**Fig. 1:**
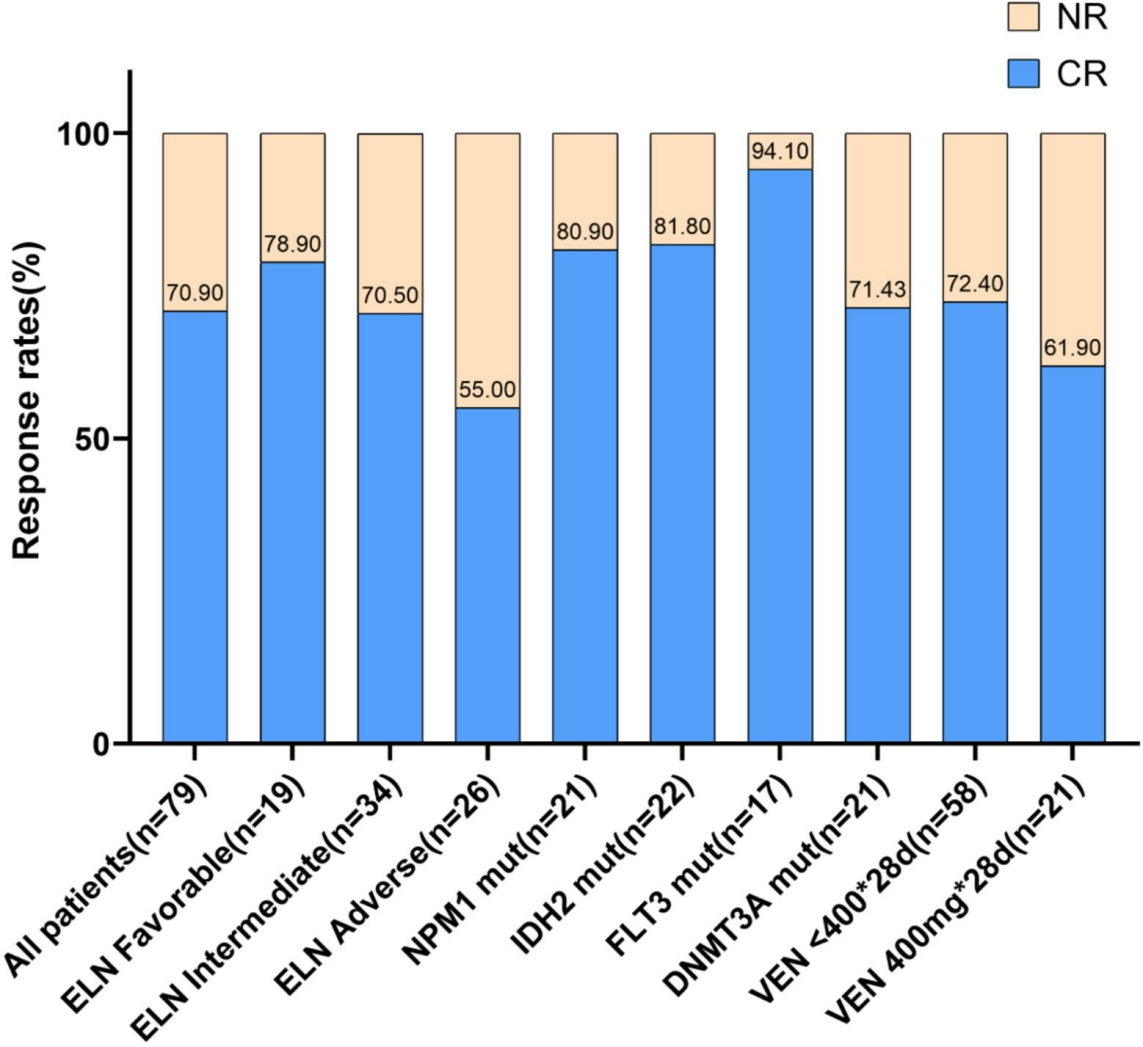
Histograms showing responses after up to at least 6 cycles.

Overall, for induction therapy, (Composite Complete Remission Rate) CRc rate was 86.4% (57/66) in AZA + VEN group and 92.3% (12/13) among patients receiving VEN + AZA combined with chemotherapy. The median time to first response (CRc) was 0.8 months (range: 0.5–1.9). Among patients achieving CRc after one cycle of VEN + AZA induction, 32 of 57 were evaluated for minimal residual disease(MRD), and 71.8% achieved multiparameter flow cytometry negativity (MRD˗), with a median time to first MRD˗ of 1.5 months (range: 1–2.6). After at least six cycles of therapy, 70.9% (56/79) of patients remained in CR/CRi, with a median duration of response (DOR) of 25.4 months (range: 6–33.7; Table 3). Overall response rates across cohorts are presented in Fig. 1. According to ELN 2022 criteria, no differences in CR rate with complete recovery were observed among favorable (78.9%), intermediate (70.5%), and adverse (55.0%) risk groups. Considering molecular variables, higher CRc rates were detected with NPM1 mutation (80.9% versus 67.2%; *P* = 0.28) and IDH2 mutation (81.8% versus 66.7%; *P* = 0.27), despite being statistically non-significant. In contrast, a significant difference was identified between FLT3 mutation (94.1%) and wild type (64.5%) (*P* = 0.017). Exploratory subgroup analyses of CRc rates demonstrated no differences between patients receiving lower (72.4%) versus standard (61.9%) VEN doses (Table S1). Among responders, 23 patients (29.1%) relapsed, with a median time to relapse of 26.2 months (range: 7.8–33.7). Seventeen patients received salvage therapy, including decitabine + VEN (*n* = 5), IDH1/2 inhibitors (*n* = 4), CHG + VEN (*n* = 4), and Selinexor (*n* = 4), of whom seven patients achieved a response. Sixteen patients (69.6%) died after relapse, with a median OS after relapse of 4.5 months.

**Table 3:** Efficacy of VEN + AZA combinations in patients with acute myeloid leukemia.

Outcomes	
First cycle	*N* = 57
CRc (CR + CRi)	50 (87.7%)
Median time to first response, months	0.8 (0.5–1.9)
Median time to first MDR negative, months	1.5 (1-2.6)
After receiving at least 6 cycles	*N* = 79
CRc (CR + CRi)	56 (70.9%)
Relapse, months	
Relapse rate of patients with CRc	23 (29.1%)
Median duration of response	25.4 (6-33.7)
Survival, months	
Median overall survival (OS)	33.5 (7-36.8)
Median replase free (RFS)	26.2 (7.8–34.9)

**Fig. 2:**
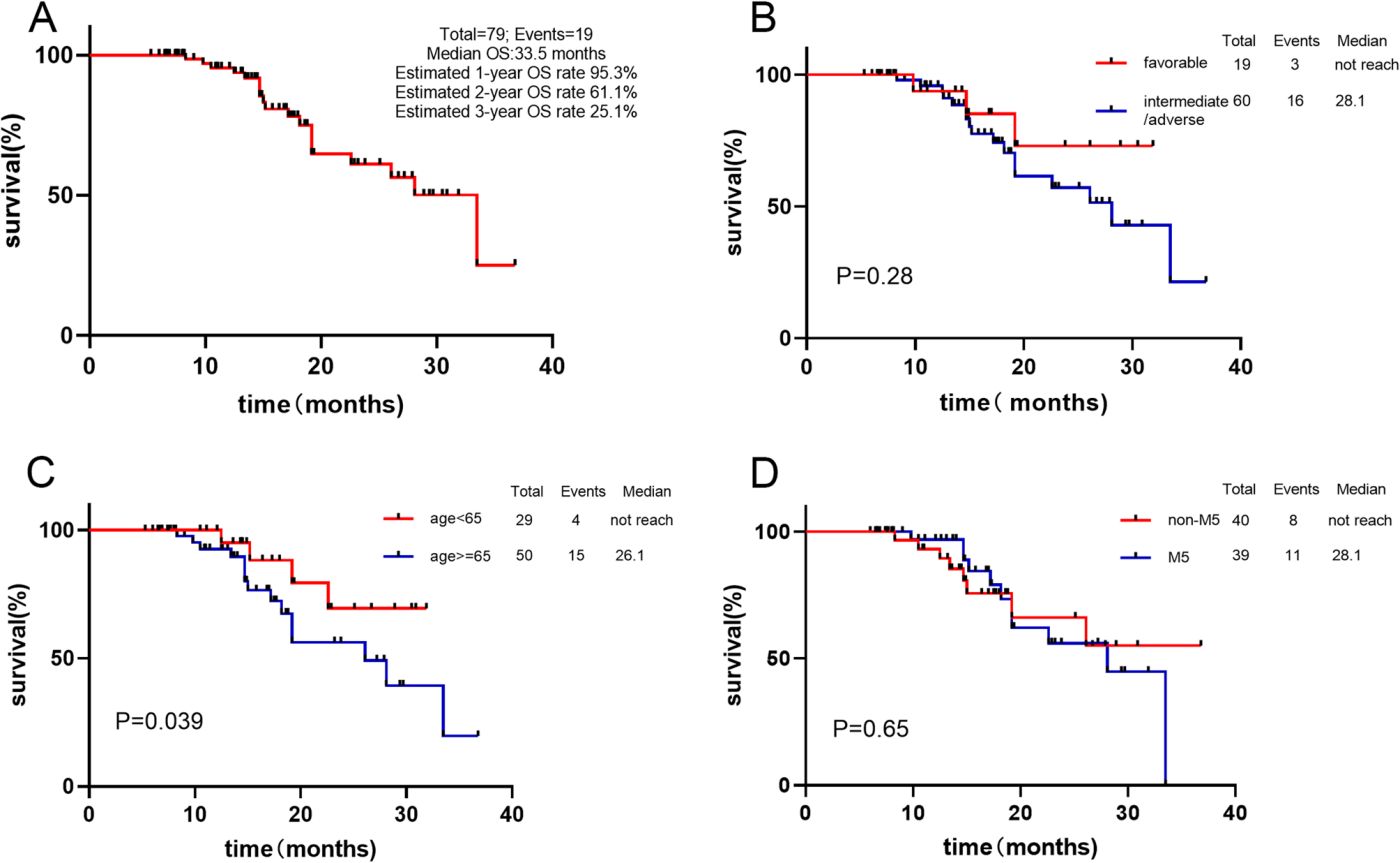
Overall survival outcomes: (**A**) For the entire group, (**B**) Stratified by ELN 2022 risk group (**C**) Stratified by age (**D**) Stratified by FAB subtype.

**Fig. 3:**
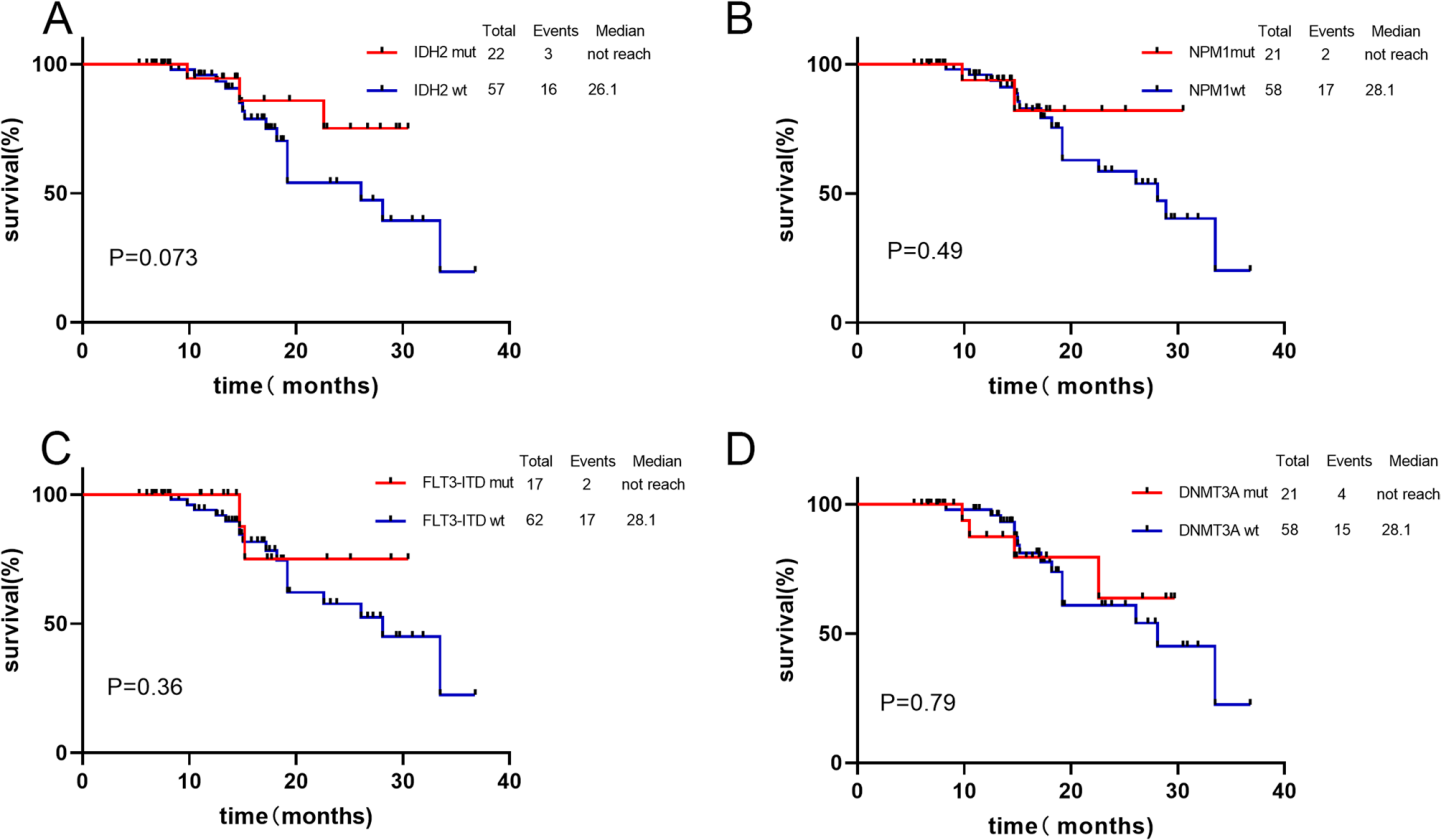
Overall survival outcomes: (**A**) Stratified by IDH2 mutation (**B**) Stratified by NPM1 mutation (**C**) Stratified by FLT3-ITD mutation (**D**) Stratified by DNMT3A mutation.

**Fig. 4:**
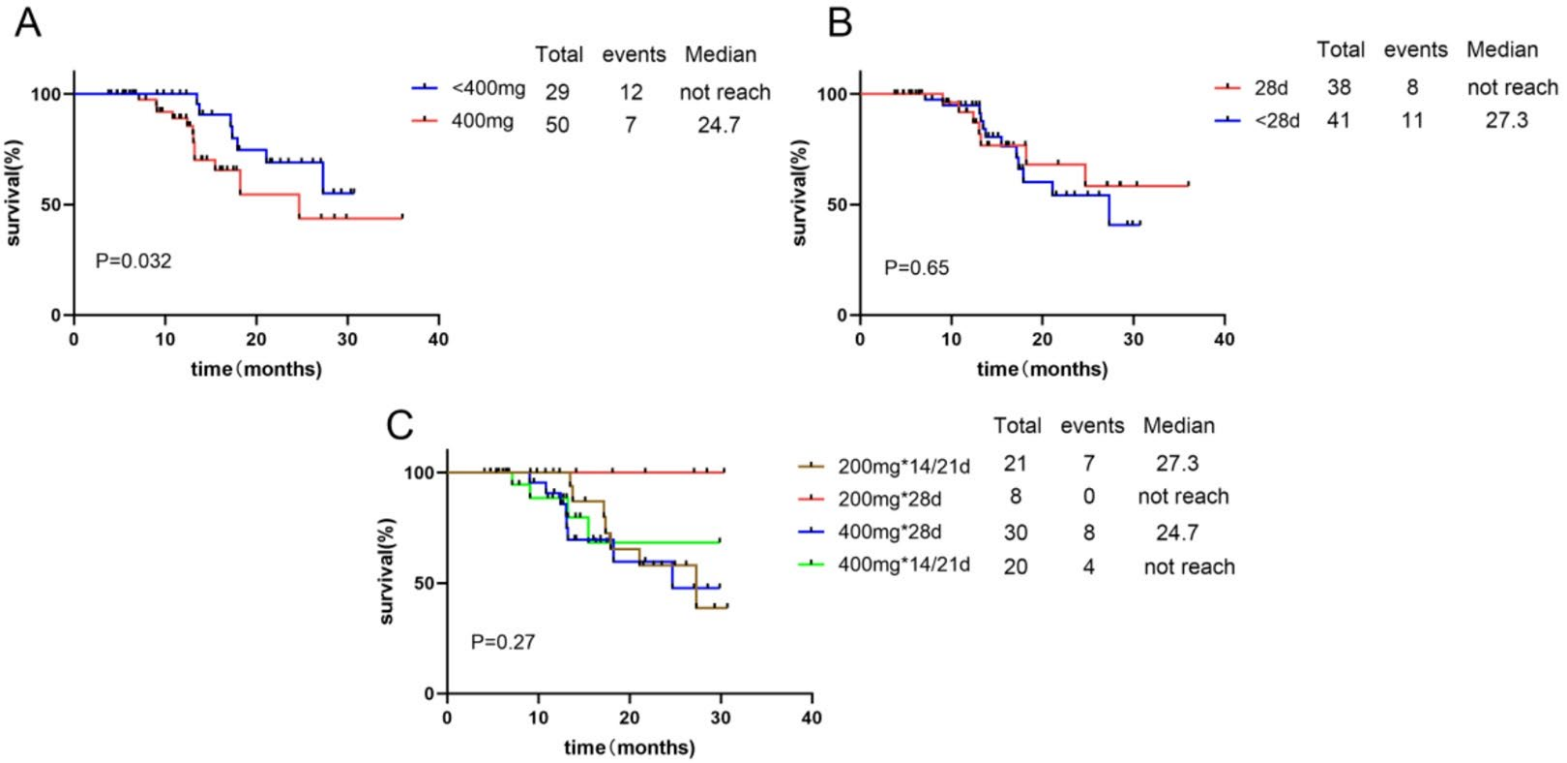
Landmark analysis of overall survival from the start of cycle 2 (**A**) Stratified by VEN dose (**B**) Stratified by VEN course (**C**) Stratified by VEN VEN dose and schedule.

With a median follow-up of 14 months (range: 6–36), the median OS for the entire cohort was 33.5 months; an estimated 2-year OS rate of 61.1% (Fig. 2A). Patients younger than 65 years demonstrated superior OS (median not reached versus 26.1 months; *P* = 0.039; Fig. 2C). Stratified by ELN 2022 risk classification, median OS was 28.1 months in the intermediate- and adverse-risk groups, while it was not reached in the favorable-risk group (*P* = 0.28; Fig. 2B). Median OS stratified by mutational status is presented in Fig. 3A–D. Patients harboring NPM1 and IDH2 mutations exhibited numerically longer median OS (not reached versus 28.1 months, *P* = 0.49; not reached versus 26.1 months, *P* = 0.073, respectively). In the landmark analysis, patients with dosage modifications(< 400 mg) experienced a significantly longer median OS compared to those without modifications (not reached versus 24.7 months ; *P* = 0.032 Fig. 4A). There was no difference in OS in patients receiving VEN < 28d days vs. 28days (Fig. 4B). Notably, patients receiving VEN 400 mg × 28 days demonstrated a numerically shorter median OS than other groups (24.7 months, Fig. 4C), although the difference did not reach statistical significance.

To further assess factors influencing survival, a Cox PH regression was performed, including age, gender, ECOG performance status, ELN 2022 risk group, WHO subtype, VEN dose/schedule, and mutational status of NPM1, IDH2, FLT3-ITD, DNMT3A, TET2, and CEBPA as variables. Univariate Cox regression analysis identified that adverse risk according to ELN 2022 was associated with an increased hazard of death (HR [2.44], 95% CI [1.09–5.48], *p* = 0.03), while age < 65 years was associated with a reduced hazard of death (HR [1.05], 95% CI [0.99–1.11], *p* = 0.046).Other variables did not reach statistical significance (Table 4). Multivariable Cox regression analysis was performed incorporating variables with *p* < 0.1 in the univariate analysis (age, ELN risk group, ECOG and FLT3-ITD mutation). In the multivariable model, none of these variables demonstrated a significant impact on OS (Table 5).

**Table 4:** Univariate analysis for Overall Survival and Relapse-free survival.

Variables	Overall survival		Relapse-free survival	
	*p*-value	HR (95% CI)	*p*-value	HR (95% CI)
Age ( < = 65 vs. > 65 )	0.046	1.05(0.99–1.11)	0.30	1.03(0.96–1.03)
Gender (Male vs. Female)	0.16	0.55(0.23–1.28)	0.57	0.76(0.29–1.96)
ECOG( <2 vs. > = 2)	0.08	1.51(0.93–2.43)	0.23	1.48(0.77–2.86)
FAB (M5 vs. others)	0.82	0.91 (0.41–2.04)	0.92	0.95(0.37–2.43)
ELN2022 ( Favorable/Intermediate vs. adverse	0.03	2.44(1.09–5.48)	0.72	1.19(0.44–3.18)
VEN schedule(< 28d vs. 28d)	0.95	1.0(0.94–1.06)	0.55	0.97(0.91–1.04)
VEN dose (< 400 mg vs. 400 mg)	0.21	1.3(0.86–1.98)	0.10	1.48(0.92–2.39)
IDH2 mutation (Presence vs. Absence)	0.59	1.25(0.53–2.95)	0.16	0.41(0.11–1.44)
FLT3-ITD mutation (Presence vs. Absence)	0.09	0.17(0.02–1.32)	0.37	0.52(0.11–2.22)
NPM1 mutation (Presence vs. Absence)	0.17	0.43(0.12–1.46)	0.65	0.75(0.21–2.26)
DNMT3A mutation (Presence vs. Absence)	0.96	1.02(0.41–2.58)	0.77	0.85(0.27–2.59)
TET2 mutation (Presence vs. absence)	0.31	0.35(0.04–2.67)	0.71	0.67(0.08–5.11)
CEBPA (Presence vs. absence)	0.41	2.33(0.30–17.9)	0.55	1.84(0.24–14.1)

**Table 5:** Univariate analysis for Overall Survival.

Variables	*p*-value	HR (95% CI)
Age (< = 65 vs. > 65 )	0.51	1.02 (0.96–1.08)
ECOG ( <2 vs. > = 2)	0.23	1.53 (0.76–3.09)
ELN2022 (Favorable/Intermediate vs. adverse	0.87	1.08 (0.41–2.94)
FLT3-ITD mutation (Presence vs. absence)	0.41	0.52 (0.11–2.43)

## Discussion

The introduction of VEN-based regimens represents a paradigm shift in the therapeutic approach for elderly or unfit patients with AML^5^. However, with the increasing utilization of VEN + AZA, critical questions have emerged regarding long-term efficacy, prognosis, optimal dosing strategies, and the feasibility of VEN administration. This commentary outlines key insights derived from administering at least six cycles of VEN + AZA in routine clinical practice. After a median follow-up time of 14 months, CR/CRi rate exceeded 70%, with a median DOR of 23.5 months, aligning with the Phase 1b study by Daniel A. Pollyea, which reported a CR/CRi rate of 71% and a DOR of 21.5 months^6^. The outcomes of patients who relapsed after AZA + VEN remained poor. Only 7 of 17 patients (41%) achieved a response to salvage therapy, with a median OS after relapse of 4.5 months, consistent with previous reports^7,8^. CR/CRi rates were the highest among patients with FLT3 (93.7%), NPM1 (80.9%), and IDH2 (80%) mutations. Moreover, median OS was numerically longer in patients harboring NPM1, IDH1, or FLT3 mutation. Previous studies have reported variable CR/CRi rates with VEN-based therapy in FLT3-mutated AML, ranging from 42.0% to 66.7%^9–11^. In contrast, CR/CRi rate in our cohort was the highest among patients with FLT3 mutation, consistent with prior findings and suggesting that VEN-based therapy may mitigate the adverse impact of FLT3 mutation^12^. The favorable prognosis observed in IDH2-mutated patients (CR/CRi 80% and median OS not reached) paralleled a pooled analysis of IDH1/2-mutated cases treated with VEN + AZA, where CR/CRi rate for IDH2-mutated patients reached 86% and median OS was also not reached. These findings corroborate preclinical evidence that 2-hydroxyglutarate-mediated COX inhibition in IDH1/2-mutant AML cells enhances BCL-2 dependence, thereby increasing sensitivity to VEN^13^. Consistently, findings from this study indicated that patients with AML harboring NPM1 mutations treated with VEN + AZA achieved superior response rates^14–16^. These high response rates observed aligned with preclinical evidence demonstrating pronounced sensitivity of NPM1-mutant AML cells to BCL-2 inhibition by VEN^17^.

The currently approved dosing schedule of AZA + VEN, comprising 7 days of AZA with 28 daily doses of VEN at 400 mg, results in severe myelosuppression, with most patients requiring VEN dose adjustments or schedule modification to manage cytopenias^2^. In this study, more than half of the patients underwent modifications to VEN dosing or treatment schedules during therapy. Similar findings have been reported in several retrospective analyses^18–21^. Although early-phase clinical trials testing VEN + AZA combinations in AML established the maximum tolerated dose of VEN, the optimal VEN duration during prolonged therapy has not been systematically evaluated and remains undefined. A previous study reported that a VEN dose of 100 mg per day for 14 days achieved CR/CRi rates and OS comparable to those in clinical trials^22^. A Mayo Clinic retrospective analysis of patients receiving AZA + VEN with varying treatment durations found similar CR/CRi rates in patients who were treated with VEN for 14 (68%), 21 (66%), and 28 days (62%), with corresponding MRD-negative rates of 65%, 72%, and 81%^20^. A recently published study also demonstrated that shortening VEN exposure to 14 days reduced the risk of complications while maintaining CR/CRi rate and survival comparable to 28-day VEN^23^. In a French multicenter study, limiting VEN exposure to 7 days produced response rates comparable to standard 28-day administration, and dose reduction of VEN appeared associated with favorable outcomes in responders^24^. Consistently, patients in this practice who underwent VEN dose reductions achieved a significantly longer median OS compared with those without modifications. However, given the limited sample size and the absence of baseline characteristics comparison between these subgroups, we cannot exclude the possibility that the favorable outcomes may be partly attributable to a higher prevalence of genetic mutations associated with better treatment response, a prospective study with balanced groups and rigorous adjustment for genetic risk factors is needed to validate these observations.

This study has several limitations. First, its retrospective design did not eliminate the potential influence of unmeasured confounding variables on the findings. Second, some subgroup comparisons were constrained by small sample sizes, preventing evaluation of differences within specific molecular and cytogenetic subsets. Third, the absence of standardized MRD data remained a limitation, as MRD has been demonstrated to be a key predictor of remission duration. Fourth, salvage therapies administered after disease progression were heterogeneous, reflecting real-world clinical practice but potentially influencing post-relapse outcomes.

## Conclusion

The combination of VEN with AZA achieved high complete remission rates and long-term OS benefit in newly diagnosed patients with AML who were ineligible for intensive chemotherapy. This analysis suggests that specific genetic mutations, such as NPM1, IDH2, and FLT3, may serve as predictive markers of treatment response, underscoring the potential for individualized therapeutic strategies. Modification of the VEN schedule and dose was associated with longer treatment duration and prolonged OS, emphasizing the importance of optimal VEN management for improved patient outcomes. Future studies with larger sample sizes are warranted to validate these findings.

## Electronic Supplementary Material

Below is the link to the electronic supplementary material.

Supplementary Material 1

## Data Availability

The data presented in this study are available on request from the corresponding author.

## Declarations

### Implications for Practice

This study represents long term experience with venetoclax plus Azacitidine for newly diagnosed patients with acute myeloid leukemia (AML) in China. Venetoclax dose changes and schedule modifications were associated with longer treatment duration and prolonged overall survival, emphasizing the importance of optimal VEN management for improved patient outcomes.

## References

[1] Shimony, S., Stahl, M., Stone, R. M., Acute Myeloid, L. & 2025 Update on diagnosis, risk-stratification, and management. *Am. J. Hematol.* **100** (5), 860–891 (2025). 39936576 10.1002/ajh.27625 PMC11966364

[2] DiNardo, C. D. et al. Azacitidine and venetoclax in previously untreated acute myeloid leukemia. *N Engl. J. Med.* **383** (7), 617–629 (2020). 32786187 10.1056/NEJMoa2012971

[3] Pratz, K. W. et al. Long-term follow-up of VIALE-A: Venetoclax and azacitidine in chemotherapy-ineligible untreated acute myeloid leukemia. *Am. J. Hematol.* **99** (4), 615–624 (2024). 38343151 10.1002/ajh.27246

[4] Dohner, H. et al. Diagnosis and management of AML in adults: 2022 recommendations from an international expert panel on behalf of the ELN. *Blood* **140** (12), 1345–1377 (2022). 35797463 10.1182/blood.2022016867

[5] Wei, A. H., Loo, S. & Daver, N. How I treat patients with AML using azacitidine and venetoclax. *Blood* **145** (12), 1237–1250 (2025). 39316723 10.1182/blood.2024024009

[6] Pollyea, D. A. et al. Venetoclax with azacitidine or decitabine in patients with newly diagnosed acute myeloid leukemia: Long term follow-up from a phase 1b study. *Am. J. Hematol.* **96** (2), 208–217 (2021). 33119898 10.1002/ajh.26039

[7] Maiti, A. et al. Outcomes of relapsed or refractory acute myeloid leukemia after frontline hypomethylating agent and venetoclax regimens. *Haematologica* **106** (3), 894–898 (2021). 32499238 10.3324/haematol.2020.252569 PMC7927994

[8] Gangat, N. et al. Outcome of patients with acute myeloid leukemia following failure of frontline venetoclax plus hypomethylating agent therapy. *Haematologica* **108** (11), 3170–3174 (2023). 36861409 10.3324/haematol.2022.282677 PMC10620560

[9] DiNardo, C. D. et al. Molecular patterns of response and treatment failure after frontline venetoclax combinations in older patients with AML. *Blood* **135** (11), 791–803 (2020). 31932844 10.1182/blood.2019003988 PMC7068032

[10] DiNardo, C. D. et al. Venetoclax combined with decitabine or azacitidine in treatment-naive, elderly patients with acute myeloid leukemia. *Blood* **133** (1), 7–17 (2019). 30361262 10.1182/blood-2018-08-868752 PMC6318429

[11] Konopleva, M. et al. Impact of FLT3 Mutation on outcomes after venetoclax and azacitidine for patients with treatment-naive acute myeloid leukemia. *Clin. Cancer Res.* **28** (13), 2744–2752 (2022). 35063965 10.1158/1078-0432.CCR-21-3405 PMC9365380

[12] Aldoss, I. et al. Venetoclax and hypomethylating agents in FLT3-mutated acute myeloid leukemia. *Am. J. Hematol.* **95** (10), 1193–1199 (2020). 32628327 10.1002/ajh.25929

[13] Chan, S. M. et al. Isocitrate dehydrogenase 1 and 2 mutations induce BCL-2 dependence in acute myeloid leukemia. *Nat. Med.* **21** (2), 178–184 (2015). 25599133 10.1038/nm.3788 PMC4406275

[14] Abaza, Y. et al. Clinical outcomes of hypomethylating agents plus Venetoclax as frontline treatment in patients 75 years and older with acute myeloid leukemia: Real-world data from eight US academic centers. *Am. J. Hematol.* **99** (4), 606–614 (2024). 38342997 10.1002/ajh.27231

[15] Gangat, N. et al. Venetoclax and hypomethylating agent combination therapy in newly diagnosed acute myeloid leukemia: Genotype signatures for response and survival among 301 consecutive patients. *Am. J. Hematol.* **99** (2), 193–202 (2024). 38071734 10.1002/ajh.27138

[16] Gangat, N. et al. Molecular predictors of response to venetoclax plus hypomethylating agent in treatment-naive acute myeloid leukemia. *Haematologica* **107** (10), 2501–2505 (2022). 35770533 10.3324/haematol.2022.281214 PMC9521222

[17] Kuusanmaki, H. et al. Ex vivo venetoclax sensitivity testing predicts treatment response in acute myeloid leukemia. *Haematologica* **108** (7), 1768–1781 (2023). 36519325 10.3324/haematol.2022.281692 PMC10316276

[18] Chatzilygeroudi, T. et al. Real-life multicenter experience of venetoclax in combination with hypomethylating agents in previously untreated adult patients with acute myeloid leukemia in Greece. *J. Clin. Med.* **13** (2), 584 (2024). 38276092 10.3390/jcm13020584 PMC10816211

[19] Yamamoto, K. et al. Venetoclax plus azacitidine in Japanese patients with untreated acute myeloid leukemia ineligible for intensive chemotherapy. *Jpn J. Clin. Oncol.* **52** (1), 29–38 (2022). 34739075 10.1093/jjco/hyab170 PMC9242001

[20] Morsia, E. et al. Venetoclax and hypomethylating agents in acute myeloid leukemia: Mayo Clinic series on 86 patients. *Am. J. Hematol.* **95** (12), 1511–1521 (2020). 32833294 10.1002/ajh.25978

[21] Vachhani, P. et al. Venetoclax and hypomethylating agents as first-line treatment in newly diagnosed patients with AML in a predominately community setting in the US. *Oncologist* **27** (11), 907–918 (2022). 35925602 10.1093/oncolo/oyac135 PMC9632323

[22] Cui, J. et al. Reduced duration and dosage of venetoclax is efficient in newly diagnosed patients with acute myeloid leukemia. *Hematology* **29** (1), 2293512 (2024). 38095287 10.1080/16078454.2023.2293512

[23] Aiba, M. et al. Shorter duration of venetoclax administration to 14 days has same efficacy and better safety profile in treatment of acute myeloid leukemia. *Ann. Hematol.* **102** (3), 541–546 (2023). 36646889 10.1007/s00277-023-05102-y PMC9977697

[24] Willekens, C. et al. Reduced venetoclax exposure to 7 days vs standard exposure with hypomethylating agents in newly diagnosed AML patients. *Blood Cancer J.* **15** (1), 68 (2025). 40246832 10.1038/s41408-025-01269-x PMC12006504

