# Supplementary material for: Real-life multicenter experience of long-term treatment with venetoclax plus azacitidine for acute myeloid leukemia in China
Source: Sci Rep. 2026 Apr 24;16:19101. doi: 10.1038/s41598-026-50426-0 (PMC13280151; doi:10.1038/s41598-026-50426-0)
Supplement: Supplementary file 1 — Supplementary Material 1 [file 41598_2026_50426_MOESM1_ESM.docx]

**Table S1** The comparison of CR/CRi rates in different group

| Variables | CR/CRi（%） | *p*-value |
| --- | --- | --- |
| *NPM1* mut | 17（80.9） | 0.28 |
| *NPM1* wt | 39（67.2） |  |
| *DNMT3A* mut | 15（71.4） | 0.99 |
| *DNMT3A* wt | 41（70.6） |  |
| *IDH2* mut | 18（81.8） | 0.27 |
| *IDH2 wt* | 38（94.1） |  |
| *FLT3 mut* | 16（66.5） | 0.017 |
| *FLT3wt* | 40（66.6） |  |
| *VEN<400mg*28d* | 13（72.4） | 0.41 |
| *VEN 400mg*28d* | 42（61.9） |  |

****Table S2.** List of genes analyzed in this study**

| ANKRD26 | DDX41 | GATA2 | KRAS | RAF1 | SOS1 | WT1 |
| --- | --- | --- | --- | --- | --- | --- |
| ASXL1 | DHX15 | IDH1 | NPM1 | RUNX1 | SRP72 | ZBTB7A |
| ASXL2 | DNMT3A | IDH2 | NRAS | SETD2 | SRSF2 | ZRSR2 |
| BCOR | ETV6 | KANSL1 | PHF6 | SF3B1 | STAG2 |  |
| CEBPA | EZH2 | KDM6A | PML | SMC1A | TET2 |  |
| CSF3R | FLT3 | KIT | PTPN11 | SMC2 | TP53 |  |
| CTCF | GATA1 | KMT2A | RAD21 | SMC3 | U2AF1 |  |

****Supplementary Table S3. the next generation sequencing data of patients****

| \| patient number \| NGS results \| \| --- \| --- \| \| sample1 \| NPM1、IDH2 \| \| sample2 \| NPM1、TET2、KIT \| \| sample3 \| SRSF2、IDH1、STAG2、RUNX1 \| \| sample4 \| FLT3-ITD、DNMT3A、NPM1 \| \| sample5 \| IDH1、NPM1 \| \| sample6 \| undected \| \| sample7 \| undected \| \| sample8 \| undected \| \| sample9 \| undected \| \| sample10 \| FLT3-ITD、IDH2、NPM1 \| \| sample11 \| undected \| \| sample12 \| DNMT3A、NPM1、WT1、BCOR、FLT3 \| \| sample13 \| DNMT3A、NPM1、TET2 \| \| sample14 \| FLT3-ITD \| \| sample15 \| undected \| \| sample16 \| undected \| \| sample17 \| NPM1、IDH2、DNMT3A \| \| sample18 \| DNMT3A、FLT3-ITD、NPM1 \| \| sample19 \| DNMT3A \| \| sample20 \| undected \| \| sample21 \| undected \| \| sample22 \| SRSF2 \| \| sample23 \| NPM1、FLT3-ITD \| \| sample24 \| ASXL1、SRSF2、TET2、DDX41 \| \| sample25 \| FLT3-ITD、CEBPA、RUNX1、DNMT3A \| \| sample26 \| DNMT3A、IDH2、STAG2、CEBPA \| \| sample27 \| SRSF2、IDH1 \| \| sample28 \| RUNX1、SRSF2、TET2 \| \| sample29 \| SMC3、CEBPA、PHF6 \| \| sample30 \| CEBPA、NPM1 \| \| sample31 \| FLT3-ITD、DNMT3A、TET2、NPM1 \| \| sample32 \| DNMT3A、EZH2、TET2、KMT2A \| \| sample33 \| FLT3-ITD 、TP53 、WT1 \| \| sample34 \| RUNX1、DNMT3A \| \| sample35 \| undected \| \| sample36 \| undected \| \| sample37 \| IDH2 \| \| sample38 \| undected \| \| sample39 \| FLT3-ITD、DNMT3A、NPM1、ASXL1 \| \| sample40 \| FLT3-ITD \| \| sample41 \| FLT3-TKD、NPM1、KRAS 、NRAS \| \| sample42 \| FLT3-ITD、IDH2、NPM1 \| \| sample43 \| CEBPA \| \| sample44 \| undected \| \| sample45 \| DNMT3A、IDH1 \| \| sample46 \| CEBPA \| \| sample47 \| undected \| \| sample48 \| BCOR、DNMT3A、IDH2 \| \| sample49 \| FLT3-ITD、RUNX1 \| \| sample50 \| BCORL1、DNMT3A、IDH2 \| \| sample51 \| undected \| \| sample52 \| NPM1 \| \| sample53 \| undected \| \| sample54 \| undected \| \| sample55 \| NPM1 、DNMT3A、IDH2 \| \| sample56 \| undected \| \| sample57 \| undected \| \| sample58 \| IDH2、RUNX1、PHF6、SMC1A \| \| sample59 \| undected \| \| sample60 \| DNMT3A、TET2 \| \| sample61 \| undected \| \| sample62 \| undected \| \| sample63 \| NRAS \| \| sample64 \| FLT3-ITD、IDH2、NPM1、PTPN11 \| \| sample65 \| FLT3-ITD、IDH2、NPM1、SF3B1 \| \| sample66 \| KRAS、KMT2A \| \| sample67 \| DNMT3A、IDH2 \| \| sample68 \| IDH2 \| \| sample69 \| WT1 \| \| sample70 \| BCOR、DNMT3A、IDH2 \| \| sample71 \| IDH2、NPM1 \| \| sample72 \| WT1 \| \| sample73 \| undected \| \| sample74 \| undected \| \| sample75 \| FLT3-ITD、NPM1、TET2 、WT1、DNMT3A、PTPN11 \| \| sample76 \| PHF6、STAG2、IDH2、RUNX1、ASXL2 \| \| sample77 \| undected \| \| sample78 \| undected \| \| sample79 \| DNMT3A、CEBPA \| |
| --- | --- | --- | --- | --- | --- | --- | --- | --- | --- | --- | --- | --- | --- | --- | --- | --- | --- | --- | --- | --- | --- | --- | --- | --- | --- | --- | --- | --- | --- | --- | --- | --- | --- | --- | --- | --- | --- | --- | --- | --- | --- | --- | --- | --- | --- | --- | --- | --- | --- | --- | --- | --- | --- | --- | --- | --- | --- | --- | --- | --- | --- | --- | --- | --- | --- | --- | --- | --- | --- | --- | --- | --- | --- | --- | --- | --- | --- | --- | --- | --- | --- | --- | --- | --- | --- | --- | --- | --- | --- | --- | --- | --- | --- | --- | --- | --- | --- | --- | --- | --- | --- | --- | --- | --- | --- | --- | --- | --- | --- | --- | --- | --- | --- | --- | --- | --- | --- | --- | --- | --- | --- | --- | --- | --- | --- | --- | --- | --- | --- | --- | --- | --- | --- | --- | --- | --- | --- | --- | --- | --- | --- | --- | --- | --- | --- | --- | --- | --- | --- | --- | --- | --- | --- | --- | --- | --- | --- | --- | --- | --- |
